# “Because he was disgusting”: transforming relations through positioning in messenger-supported group psychotherapy

**DOI:** 10.3389/fpsyg.2023.1268989

**Published:** 2024-01-04

**Authors:** Susanne Kabatnik

**Affiliations:** Digital Humanities, Faculty II, University of Trier, Trier, Germany

**Keywords:** interactional linguistics, positioning, helping interactions, group psychotherapy, transforming relationship

## Abstract

**Introduction:**

This article deals with positioning in messenger-supported group psychotherapy in terms of transforming relations. The aim of the messenger-supported therapy format is to work through conflicts that have arisen with people via messenger services. This is achieved in different phases of conversation, such as describing the situation, analysing one's own behaviour and defining wishes, by collaboratively drafting a message to the person from the conflict.

**Methods:**

The data basis is a corpus of 14 video-recorded group psychotherapy sessions. Methodologically, the study is guided by interactional linguistics, a linguistic research field that focuses on interpersonal interaction.

**Results:**

Using a case study, I show how the interactants work through a conflict through positioning, constitute group identity and relationships, and thus also transform their stance concerning the issue. Moreover, positioning serves the collaborative formulation of a message and thus also the change of the relationship to the person from the messenger communication.

**Discussion:**

Relationship management in eSA group psychotherapy can be observed on different levels: (1) among the interactants in the room, (2) with the persons from the chat messages, and (3) between the patient(s) and the therapist.

## 1 Introduction and research context

### 1.1 eSA group psychotherapy as an innovative helping format and positioning theory

In many institutional settings, smartphones are usually perceived as a distraction. This is different in eSA group psychotherapy (“electronic Situation Analysis”), where the use of smartphones is explicitly encouraged. This innovative therapy format was developed at the LMU Munich and aims to treat chronic depression (Grosse-Wentrup et al., 2020)^1^. The concept is based on the assumption that people with depression often suffer from interpersonal problems in addition to their depressive symptomatology (Schramm et al., 2011), which manifest themselves in interpersonal interaction, i.e., verbally and in writing. With the help of smartphones in eSA group psychotherapy, the patients' conflictual messages are analysed weekly in the group, and suggestions for solutions are drafted in the form of (re)formulated and co-constructed text messages, i.e., in concrete terms: one person is selected weekly in the session to present a conflict with a friend, colleague or family member that has arisen *via* messenger service.

The aim of eSA group psychotherapy is then to formulate a message for the problem presented to bring about a change in the patient's communication and thus work on interpersonal problems. These two goals can be pursued in group psychotherapy in two ways: firstly by formulating text messages to family and friends and secondly by working on this common project. In this way, relationships are built both between group members and between patients and therapists, which is a current research focus in the field of applied linguistic research on helping interactions (Scarvaglieri et al., 2022b). As social isolation is part of the symptomatology (Bressiere et al., 2008), it is even more important to study the interaction of people with depression.

In group psychotherapies, social systems are established (cf. Preyer, 2012, p. 121). which necessarily form structures with specific structural components, such as role, status or expectation, which are both self-selected and actualised in social interaction. Groups structure themselves as social systems through their structural components as well as the determination of an ingroup and outgroup (Kabatnik, 2023a), resulting in group dynamics (Preyer, 2012, p. 121ff.). Through their function of marking persons or objects of speech as outgroups, positioning thus plays a decisive role in the formation of groups and forms one aspect of their dynamics.

In the following example, which takes up the title sequence and positioning “because he was disGUsting”, the group members discuss the conclusion of the collaboratively formulated message in group psychotherapy.

Excerpt 1 (51:07–51:39):


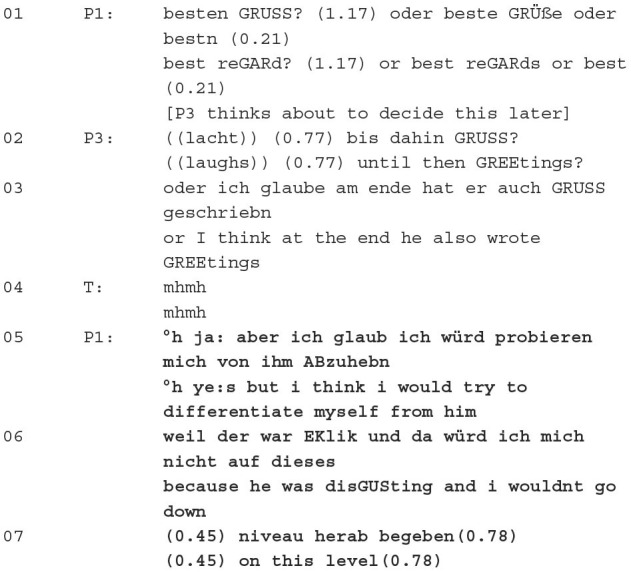


The patients consider together which formulation is most suitable. P1 suggests *best regard* or *best regards*, P3 *until then greetings* and follows up with her assumption that the professor—who will be the subject of this case study and this article—ended his message with *greetings*. P1's suggestions are not accepted. This is verbalised by a lack of acceptance of the suggestion and by P3 initially postponing this message part until later. In this way, P3 positions herself in a negative way towards the suggestions. P3 considers using the same greeting phrase as the professor. She thereby implicitly expresses that she would imitate his verbal behaviour. This is initially affirmed by P1. However, through the adversative clause °*h ye:s but i think i would try to differentiate myself from him*, a contradictory opinion is expressed, namely to stand out from the professor. P1 justifies this by the predication *because he was disGUSting and I wouldnt go down*, whereby she evaluates him and his behaviour, expresses her extreme rejection and marks him as belonging to the outgroup.

Positioning can be localised—as this example shows—on different levels, for example, to characterise people or their relationship to each other as well as to evaluate formulation suggestions of the group. In this article, positioning is examined from different perspectives: The analysis shows different points of reference and various practises for positioning.

The different positioning practises (Torres Cajo, 2022) include categorisation practises, in which speakers categorise themselves or others, for example, by means of a category label (e.g., I'm more of an Apple person); attribution practises, in which speakers attribute dispositional characteristics to themselves, for example, by predication (e.g., I'm sporty); evaluation practises, in which speakers evaluate behaviours in order to position themselves morally normatively (e.g., I think his behaviour is bad); narrative practises in which speakers position themselves through narratives (e.g., The other day I was in the shopping centre again for years); authentication practises in which speakers prove their positioning through examples (e.g., I am sporty, I have already won many sports competitions); and enactment practises in which speakers realise their positioning performatively, for example through knowledge displays (e.g., I was there, I heard him say it myself).

I will argue that various positioning practises (Torres Cajo, 2022) take on a central role between interactants in group psychotherapy, e.g., in relation to the people involved, their behaviours, and the formulations suggested by the group. Which interactive practises do the interactants use to position themselves in psychotherapy? And which function do positionings have in this helping format? These are the research questions I address in today's presentation. Because positioning is considered in this article in relation to the constitution of relationships, I begin with an outline of linguistic (interactional) research on the constitution of relationships in helping interactions. After that, I discuss my data basis and methodological approach. Then, I will analyse examples of positioning with regard to their functional aspects and discuss them in a conclusion.

### 1.2 Relationship constitution in pragmatics research and in helping interactions

The shaping of relationships is firmly anchored in pragmatics research. Through the feature of dialogicity, language in interaction is emphasised as essential in language-theoretical approaches (von Humboldt, 1963; Bachtin, 1979, 1996; Linell, 1998; cf. Mandelbaum, 2003b). For Bühler and Jakobson, interpersonal relations manifest themselves in the functions of expression, appeal, illocution, and perlocution, as well as the phatic function of speech acts, which both indicate and constitute the relationship among interactants (Jakobson, 1973/2014; Bühler, 1990). Watzlawick et al. (1967) also emphasised the relationship-constituting aspect of language in addition to the information content. Particularly fruitful concepts for the linguistic study of relational constitution in interaction come from the sociology of interaction, for example, through Goffman's (1955, 1967) remarks on role and face, as well as Brown and Levinson's (1978, 1987) politeness theory. The concept of positioning was developed by Davies and Harré (1999), which, as a social constructivist approach, focuses in particular on dynamic aspects of interpersonal relationships. According to Davies/Harré, social identity emerges through its production in discourse, whereby they assume a reflexivity of discourse and different positions (see also, e.g., Harré and Van Langenhove, 1991; Davies and Harré, 1999). Through the dynamic concept of positioning, the static, formal and ritual-focused concept of role in social interaction can be expanded (cf. Davies and Harré, 1999: 43). Role and positioning are structural components of social systems through which groups structure themselves dynamically and in interaction (Preyer, 2012). Role, positioning, and thus group dynamics can be further influenced by institutional constraints—caused by different hierarchies (cf. Magee and Galinsky, 2008, p. 351). Holly (2001) also addresses relations and describes them as elementary. They are ubiquitous, every day and mostly implicit—which makes the study of relationships difficult. Relational work encompasses the entire spectrum of interpersonal aspects of social practises (Locher and Watts, 2008). Mandelbaum (2003a, p. 217) describes relationships “as collections of communicative practises, or things that we do through communication, in contrast to thinking of them as social structural things that we have”. Bucholtz and Hall (2005) followed this by outlining a framework for the construction of identity that emerges in social interaction. Through the premise of the construction of relationships in interaction, conversation analysis can be used to analyse relationships and their construction (Sidnell and Stivers, 2013). In successive sequences, interactants constitute linguistic actions, action goals, and relationships (Kabatnik et al., 2022). In helping interactions (Graf et al., 2019), it is precisely this constructional character of interpersonal interaction that is elementary. Because of the asymmetrical constellation of help-seekers and help-receivers, the co-construction of help is essentially shaped and supported by the formation of relationships in conversation. This requires both joint interactional work and the establishment and achievement of common goals (Muntigl et al., 2012; Muntigl and Horvath, 2014; Kabatnik et al., 2022, p. 144f.). Setting up and achieving shared goals is considered a core element of successful therapy in psychotherapy (Muntigl et al., 2020). The therapeutic alliance is even postulated as the most effective success factor in psychotherapy, whereby the constitution of relationships in helping interactions is the best and most reliable predictor of desired psychotherapeutic change and calls for appropriate research intensity (see also Horvath and Greenberg, 1994; Horvath, 2006; Ardito and Rabellino, 2011; Flückiger et al., 2012; Lambert, 2013; cf. Ribeiro et al., 2013, p. 295). Scarvaglieri et al. (2022a), for example, dedicate an entire anthology to the shaping of relationships in helping interactions, in which different helping formats are examined from the point of view of the co-construction of relationships, e.g., doctor–patient conversations (Džanko, 2022; Günthner, 2022; Kuna and Scarvaglieri, 2022; Thurnherr, 2022), psychotherapy (Buchholz, 2022; Guxholli et al., 2022; Kabatnik et al., 2022; Muntigl, 2022; Pawelczyk and Faccio, 2022), coaching (Graf and Jautz, 2022; Winkler, 2022), as well as newer helping formats in the social web, such as support through illness-related forums in social media (Kabatnik, 2022). The research focuses there, for example, on the constitution of a sense of community between psychotherapist and patient (Buchholz, 2022), the face-threatening question *What about you* in psychotherapy (Guxholli et al., 2022) or semi-responsive answers (Winkler, 2022). What these studies have in common is that mostly only dyadic helping formats and the constitution of relationships between professionals and clients are examined.

The present article joins this tradition of interactional linguistic research on the formation of relationships in helping interactions by examining positioning practises. It complements this focus of research with a study on messenger-supported group psychotherapy. Analysing therapeutic group interaction (instead of dyadic communication) by focusing on positioning practises from an interactional linguistics perspective is what is new and innovative in the field of helping interactions.

## 2 Materials and methods

The data basis for the study is a large corpus of 14 videotaped group psychotherapy sessions. These were recorded between October 2021 and October 2022 at the Department of Psychiatry and Psychotherapy (LMU Munich). The sessions have an average length of about 1 h. Thus, the collected video material totals about 14 h and 43 min.

**Figure 1:**
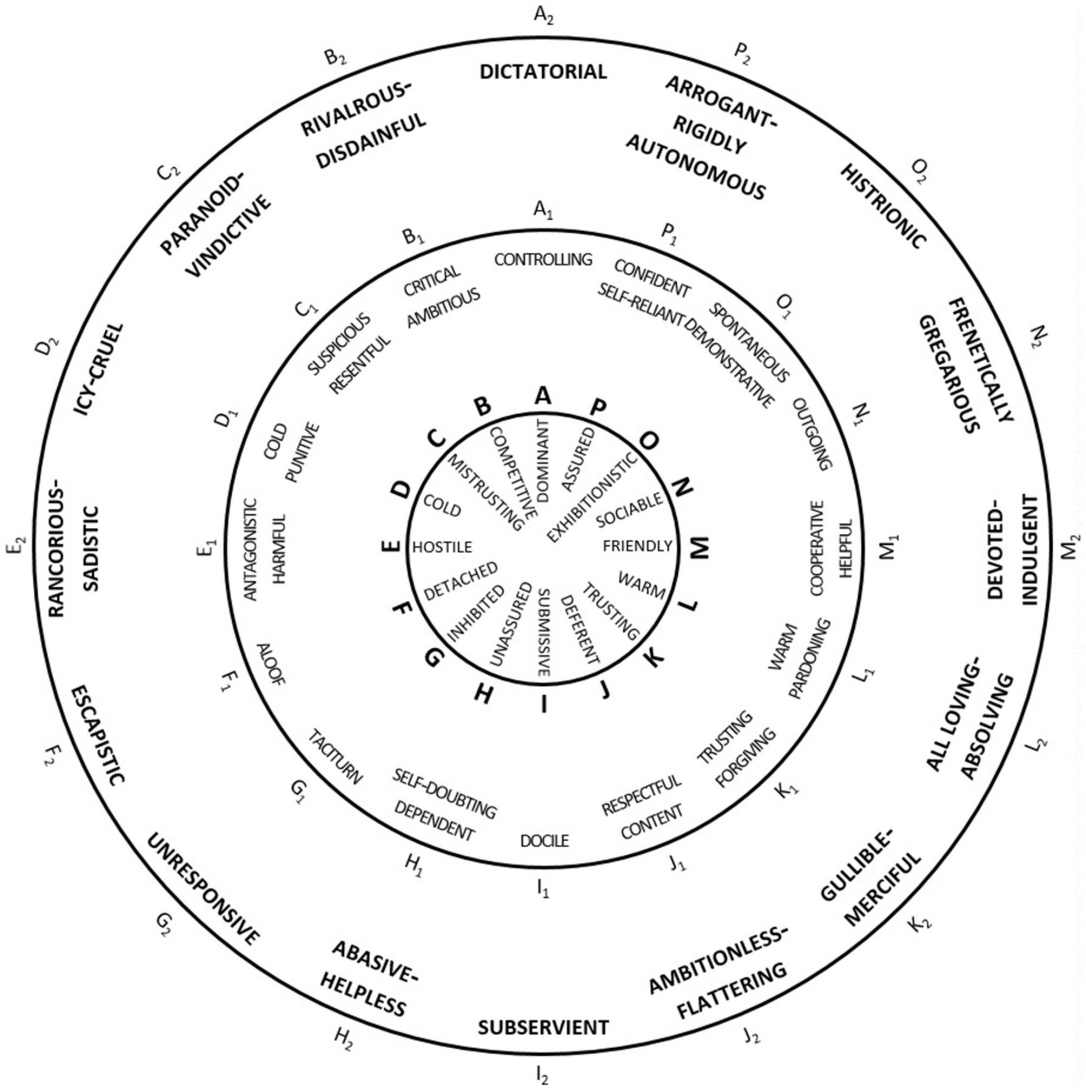
The Kiesler (1983).

The messenger-based therapy format takes place weekly on a voluntary basis. The sessions are led by one to two therapists (1 T = male, 1 T = female). In the whole data material, there are 30 different patients (8 P = male, 22 P = female). In addition to the patients and therapists, in the room, there is a flipchart and a poster of a psychotherapeutic instrument, the Kiesler circle (see Chapter 3 and Figure 1). Important intermediate results and the draught message are written down on the flipchart. The poster with the Kiesler circle is used by the interactants to evaluate their behaviour, goals, or wishes.

For the analysis of interactive practises for positioning, I will take a closer look at one video recording of one group psychotherapy session (minimum transcription according to GAT2, Selting et al., 2009). The session has a total length of about 57, 56 min. Three patients and a therapist are present, as well as a trainee who records the conversation. The three patients are female and aged between 27 and 43 years at the time of admission (P1 was born in 1983, P2 in 1977, and P3 in 1993). All three are patients with chronic and/or recurrent depression who have been in the cognitive behavioural analysis system of psychotherapy (CBASP) programme (McCullough Jr et al., 2014) and also in the electronic Situation Analysis (eSA) group psychotherapy (Grosse-Wentrup et al., 2020) for 4–6 weeks together (although with changing members). The CBASP approach is based on the assumption that people with recurrent or chronic depression have not (sufficiently) learned to verbalise needs, wishes, and thus positioning in social interaction due to traumatic experiences in childhood. Traumatic experiences cause patients to transfer (mostly unconsciously) (repressed) emotions, reactions, and expectations but also wishes or fears to new social relationships (transference hypothesis). In order to correct their negative-depressive assumptions about life in other experiences, people with depression have to learn that different people can react differently in the same situation (cf. Schramm et al., 2011). This is accompanied by a subjectively experienced low ability to act, depressive thoughts, and social isolation (cf. Brakemeier et al., 2012, p. 6ff.), which not only justifies but also makes a (conversational) linguistic examination of the positioning of people with depression relevant. The original data are in German but were translated into English for this article.

The research method is interactional linguistics (Imo and Lanwer, 2019). Interactional linguistics studies language in interaction and takes the participants' perspective to analyse their mutual understanding from the (sequentially) next turns and utterances. Thus, the focus is on interactional language use, which is characterised by its sequentially structured, collaborative, and situation-based construction of meaning and structure (Imo and Lanwer, 2019, p. 2). In this way, conversations fundamentally rely on sequentiality, i.e., successive utterances, in verbal interaction (Deppermann, 2008). Psychotherapy is a verbal and co-constructed treatment format that relies on structural features of communication. Through the sequence of utterances by (at least) two interactants, intersubjectivity is established in (psychotherapeutic) conversation, i.e., through the exchange of knowledge and positions, a common knowledge base emerges among the interactants (e.g., Heritage, 2012). This shared knowledge base then forms the basis for therapeutic effectiveness and relationship building (Peräkylä et al., 2008; Kabatnik et al., 2022).

## 3 Analysis

The group psychotherapy can be divided into different phases of the conversation (Kabatnik u.r.). I derived the phases of the conversation in an inductive and deductive analysis process. The deductive derivation procedure is based on the given structuring of the psychotherapy according to the CBASP approach. Following this approach, the therapy is initially divided into an analysis phase and a solution phase. The analysis phase of eSA group psychotherapy includes the following steps: description of the communication, interpretation of the other person's message, characteristics of the message, actual outcome, desired outcome, and comparison of the actual outcome with the desired outcome. The solution phase follows the revision of the interpretation and the change and reformulation of the message.

The inductive derivation of the phases ensued from the data set by analysing the transitional formulations. Each phase is introduced by the therapist with a transitional formulation, such as in phase 4 (see Table 1). The analysis of one's own behaviour through *if you look at your (.) OWN behavior (1.28) so (.) [repeated behavior] where would you place yourself in the KIESler circle?* (17:30–19:13). From the linguistic analysis of the transitional formulations, a classification of the conversation phases into 10 phases results, which partly overlap with the structure inherent in the psychotherapy format. The following 10 conversation phases could be identified as follows: the phase of greeting, the definition of the session goals, the description and interpretation of the situation, the analysis of one's own behaviour and the actual result as well as the desired result, followed by the formulation phase, the comparison with the desired result, followed by a final reflection on the session, and the conclusion of the discussion (see Table 1).

**Table 1:** Speech phases in eSA group psychotherapy.

**Phase**	**Description**	**Timecode**	**Introduction**
1	Greeting	01:23–01:44	T: exactly (.) nice that you are THERE (.) in our little ROUND,
2	Definition of the session goals	01:44–02:50	T: what are your PLANS for today?
3	Description and interpretation of the situation	02:50–17:30	T: what kind of situation IS it?
4	Analysis of one‘s own behaviour	17:30–19:13	T: if you look at your (.) OWN behavior (1.28) so (.) [repeated behavior] where would you place yourself in the KIESler circle?
5	Actual result	19:13–20:36	T: if you look now (.) ACTual reSULt? °h (0.23) how did you SHAped the relationship (.) h° by your behavior? how did it turn OUT for you?
6	Desired result (ca. 10 min Ventilation break)	20:36–38:57	T: okay (.) and what would you have liked (.) for the situation to turn OUT like? (1.36) how would you like to have behaved
7	Formulation phase	38:57–52:50	T: then we get down to formuLATing;
8	Comparison with the desired result	52:50–53:57	T: where did you end up in the KIESler circle?
9	Final reflection on the session	53:57–57:05	T: then we go into the FInal round:,
10	Conclusion of the discussion	57:05–57:37	T: then MANY thanks, all of them, (.) [Farewell]

The solution phase in the psychotherapeutic concept corresponds to the formulation phase in the linguistic analysis. A further subdivision of the solution phase based on linguistic features could not be observed. The phases of conversation produce affordances for positioning, i.e., possibilities for evaluations and the expression of attitudes are already inherent in the conversational format. In the phase of describing the situation, for example, the speakers can position themselves in relation to objects or persons (Chapter 3.1). Further possibilities for positioning are inherent in the phase of classifying the speaker's own behaviour in the Kiesler circle (Chapter 3.2). The Kiesler circle (Figure 1) is a psychotherapeutic instrument for classifying feelings and behaviours, i.e., it is used for positioning or determining them and physically hangs as a poster in the group psychotherapy room. The concept was developed by the US psychologist Donald Kiesler in 1983 (cf. Guhn and Brakemeier, 2022). Kiesler (1983) assumed that difficulties in social interaction can be described on two axes, namely firstly, the axis with the opposite poles dominant/open and submissive/closed and secondly, the affiliation or relationship axis with the opposite poles friendly/close and hostile/remote, including mixed forms such as friendly dominant or submissive–hostile (cf. Kiesler, 1983, p. 186f.). This diagram can then be used to classify—especially communicative—behaviour, i.e., to position oneself in relation to it. The Kiesler circle training aims at adapting actual behaviour to the desired behaviour depending on the situation (cf. Guhn and Brakemeier, 2022). The formulation phase (Chapter 3.3) opens up further space for positioning—the group is required to decide together which formulation is suitable for the goal that has been set.

In the following, I will start with the analysis of the description phase in which Patient 3 presents the conflict to be discussed and positions herself in her description.

### 3.1 Description of the situation

In the phase of describing the situation, a patient from the group psychotherapy presents a conflict that has arisen *via* the messenger service. In doing so, the person presenting has to bring the group to the same level of knowledge (e.g., cf. Spranz-Fogasy et al., 2018; Kabatnik, 2023b). In the case study chosen for this article, Patient 3 presents her conflict with a professor, which is caused by the professor's use of an incorrect and insulting form of address in an email to the patient. Different positionings are verbalised in the description itself and in the reactions to it, which play a decisive role in the further interaction in the group psychotherapy.

Excerpt 2 (02:53–09:34)—Description of the situation


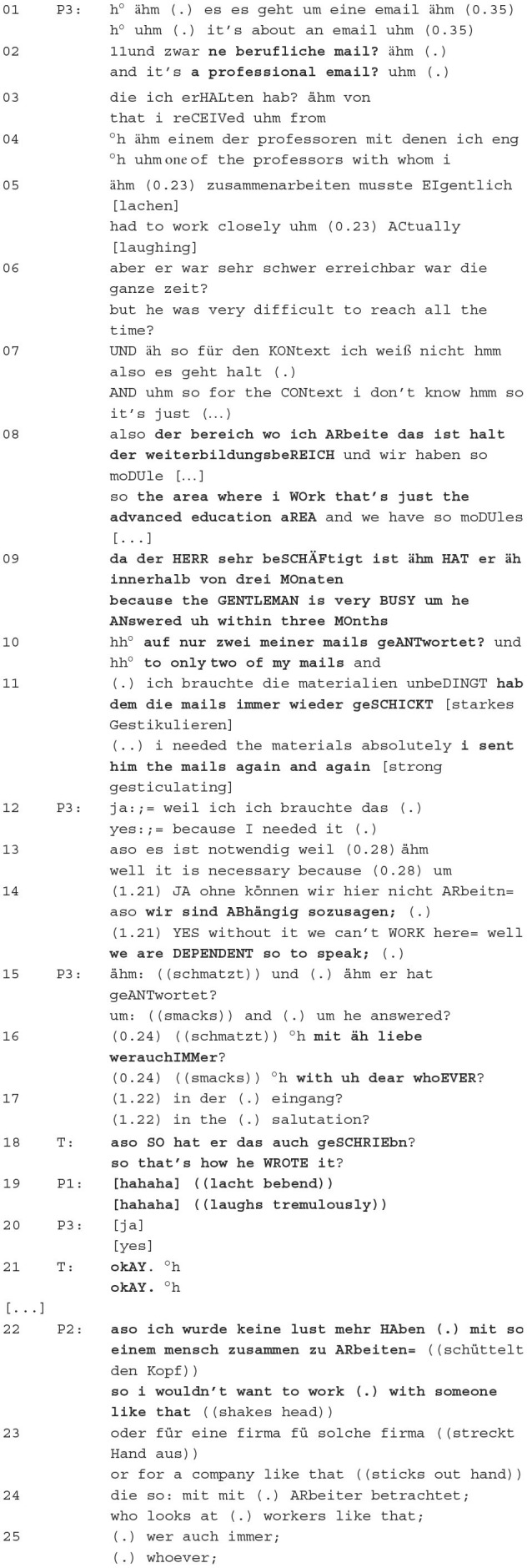


Patient 3 describes the conflict situation for the group. She begins by contextualising the (conflict) situation, describing the type of correspondence and the relationship with the person from the messenger communication, i.e., the allocated professor. She continues with a brief description of the problem, namely the (in)availability of the professor for upcoming common tasks. P3 then goes into more detail about her professional situation in the university context and repeats the problematic contact with the professor. This is followed by justifications for her multiple contacts with him—she absolutely needed materials from him and could not continue working without them, i.e., she was dependent on his input. She then reads out the message with the inadequate salutation “dear whoEVER”.

Regarding the positionings in this excerpt, P3 first classifies the situation categorically, namely that it is a professional email, not a private one and that she works in a university context. The utterances *it's a professional email* and *the area where i work that's just the advanced education area* from the narrative of Patient 3, which functions here as a categorisation practise (Torres Cajo, 2022, p. 65/70) and serves to classify the conflict for the group in comparison to other conflicts, for example, with family or friends. This classification by the patient activates specific knowledge in the group members, through which the patients can access their knowledge about professional (conflict) situations and provide adequate formulation suggestions in the further course of the conversation (see Chapter 3.3).

The patient then goes on to describe how she had a hard time reaching the professor, so that the expression *since the gentleman is very busy, he has replied to only two of my emails in 3 months* can be classified as another positioning practise. Through the ironic *gentleman*, Patient 3 socially categorically ranks the professor higher. The thematisation of his poor email correspondence serves moral-normative blame as an evaluative practise (Torres Cajo, 2022, p. 142ff.). By addressing the professor as a gentleman and informing the group about her communication behaviour with the professor, Patient 3 implicitly verbalises her relationship with him. She ranks him higher in the hierarchy and, at the same time, ridicules this hierarchical higher ranking through the expressed irony. Thus, P3 marks her and the professor's positions as asymmetrically in terms of institutional roles (“he above her”) by emphasising her professional dependence on him as well as in terms of morality (“she above him”) by ridiculing him.

The asymmetrical relationship constellation between her and the professor is also expressed by the professor's writing behaviour. This is because he does not reply to the patient—without any institutional consequences—which can generally be interpreted as impolite, potentially face-threatening and relationship-destructive behaviour (cf. Simmons, 1994). Thus, the evaluation practise here functions not only as a moral-normative assessment of his behaviour but also as a characterisation of the patient's relationship with the professor.

She goes on to say that she tried to contact him several times (*i sent him the mails again and again*) as a narrative practise (Bamberg, 1997; Lucius-Hoene and Deppermann, 2004; Georgakopoulou, 2007; cf. Torres Cajo, 2022, p. 158ff.). By displaying repetitive behaviour, the patient shows her effort to get in touch with the professor. She thus positions herself as very engaged, which is expressed by the iterative *again*. She concludes the narrative by saying that she was absolutely dependent on his help, (*we are dependent*), describing her relationship with the professor through predication as dispositionally dependent, i.e., as an attributional practise (Torres Cajo, 2022, p. 108ff.). The patient then reads out the professor's salutation, namely *Dear WhoEver*, in a professional email, which is realised by the patient with numerous hesitation markers. In this way, she expresses the delicacy of the topic (Spranz-Fogasy et al., 2023).

The therapist reacts here with *so that's how he WROTE it? (*‘*aso SO hat er das auch geSCHRIEbn?*'*)*. She marks her surprise about the professor's formulation by using *so* “*aso*” (see, e.g., Golato and Betz, 2008) and the question *that's how he wrote it?* Through the formal question, the therapist, on the one hand, assures her understanding and, on the other hand, expresses her bewilderment, through which she evaluates the professor's behaviour and positions herself in this way. This could be seen as an affiliative utterance towards the patient. Muntigl and Scarvaglieri (2023) state, that “[a]ffiliation can be understood as trust, commitment and intimacy […] and is related to the emotional agreement and the bond […] created in interaction. Patient 1 reacts with trembling laughter, which also expresses her position towards this form of address: She considers this behaviour too extreme (cf. Glenn, 2003, p. 112ff.). Patient 2 voices her unwillingness to work for such a person or company, taking herself as an example, i.e., she would no longer want to work for the professor in place of Patient 3 as a performative positioning practise (Torres Cajo, 2022, p. 194ff.).

According to her role in the session, P3, as a patient with the messenger conflict, has to mark an outgroup person. Through the patient's description with different positionings, Patient 3 shows how she relates to the professor and evaluates him and his behaviour. This evaluation is understood by the interactants, and they side with the patient through their reactions and thus express their first solidarity. Furthermore, following the group dynamics, the group members have to reaffirm or repeat this marking afterwards. This is expressed here by the further positionings.

The therapist has a key role in this process: Because of her role and status in the conversation, she has the function of guiding and structuring the conversation (cf. Marciniak et al., 2016, p. 4f.). This is also revealed by the sequential order of the utterances since the therapist has the right to speak directly after P3's description. Her positioning on the conflict situation is decisive here. In a case of contradiction on the part of the therapist, subsequent positioning could deviate from P3's stance. This comparison makes it clear that the therapist's positioning is crucial for the subsequent interaction and the group-building process.

### 3.2 Classification of behaviour in the Kiesler circle

In the phase of classification of the patient's behaviour in the Kiesler circle, the conflict is viewed and classified from the perspective of the patient involved. He/she is supposed to evaluate and classify his/her own behaviour. In this phase, positionings are requested, which are crucial for the subsequent formulation phase and are presented in the example of the Excerpt 2.

**Excerpt 3 (17:38**–**18:57)**—**Classification of behaviour in the Kiesler circle**


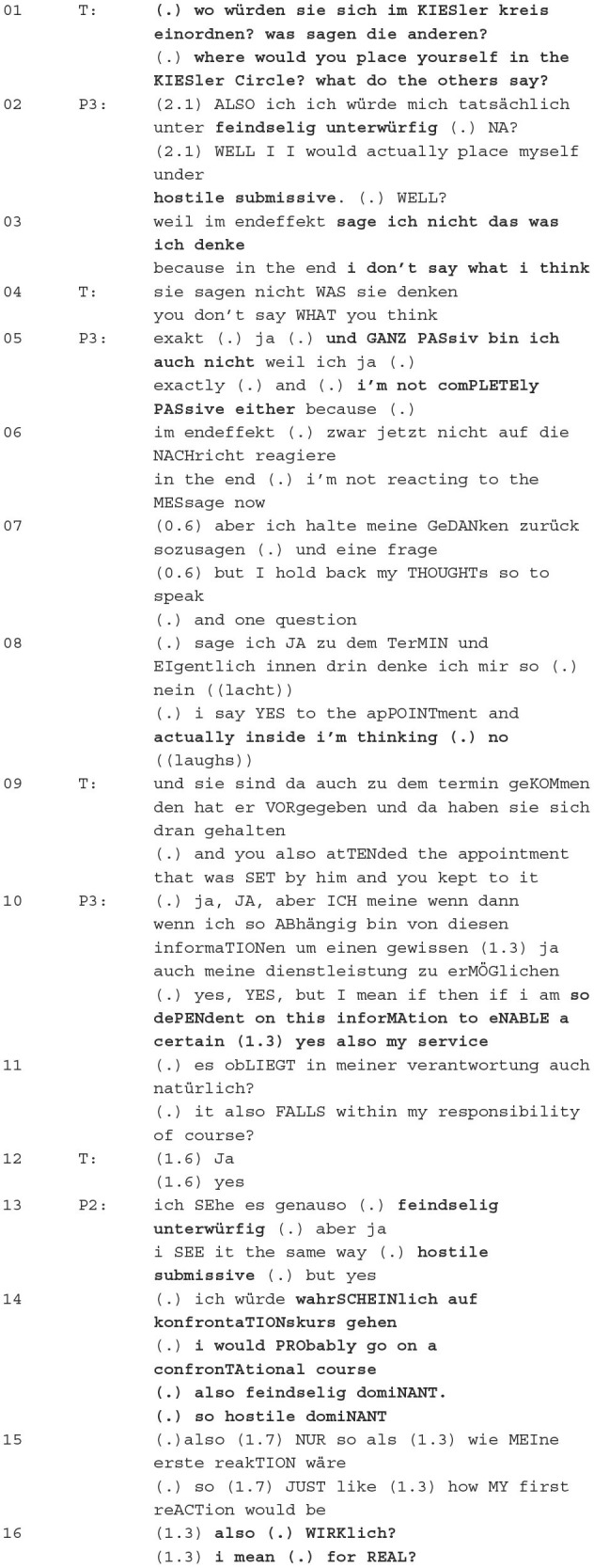


The therapist introduces the phase with “*Where would you place yourself in the Kieser Circle? What do the others say?*”, thereby eliciting a positioning of Patient 3 and the whole group. P3 classifies herself as hostile and submissive in the Kiesler circle, thereby evaluating her own behaviour (evaluation practise; Torres Cajo, 2022, p. 142ff.). She justifies her classification by the fact that she does not express any positioning towards the professor. She further evaluates her behaviour because she is not entirely passive either (attribution practise; Torres Cajo, 2022, p. 108ff.). The ambivalence in her behaviour can be seen because she actually wants to come to the Zoom meeting out of a sense of duty, but *actually inside [she is] thinking (.) no*. She has come to the meeting because she is in a relationship of dependency with the professor and wants to make her service possible, which represents a categorical classification through the institutional context at the university (professor vs. assistant) and constitutes the core of the conflict: She wants to position herself towards him, but cannot due to the dominant relations of power.

P2 then takes another positioning of P3 by confirming her classification as hostile and submissive (evaluation practise; Torres Cajo, 2022, p. 142ff.) and then suggests what she would do, which is probably to go confrontational, and classifies her affect as hostile–dominant, thereby evaluating her hypothetical behaviour (evaluation practise; Torres Cajo, 2022, p. 142ff.). Patient 2 concludes her utterance with *I mean for real?*, thus repeatedly referring to the professor's salutation and verbal behaviour and again expressing her negative evaluation. Such positioning, which evaluates the professor or his behaviour, is found numerous times in this conversation, such as the title-giving *because he was disgusting*. In this way, the whole group opposes the professor and supports the patient, which has group identity-forming and relationship-constituting functions (Deppermann and Schmidt, 2003, p. 25ff.).

The therapist ratifies P3's positioning by repeating her explanation, through which the therapist implicitly agrees with P3. In this way, she supports the patient in classifying her behaviour in the Kiesler circle and thus lays an important milestone in the process of change. The positionings here serve to establish an actual state; the patient should recognise and discuss how she has behaved verbally in order to define a desired state in the next steps, which forms the basis for the joint project of message formulation. The actual state can then be compared with a target state, namely, writing a dominant message to the professor and setting a boundary.

### 3.3 Formulation phase

The formulation phase is the phase in messenger-supported group psychotherapy in which the patients collaboratively formulate a response to the person from the conflict situation. In this phase, a text message is created that can be sent to the corresponding person at the end of the session. During this phase, numerous positionings can be found that refer either to a formulation suggestion or a behaviour, which are presented in the example of the Excerpt 3.

**Excerpt 4 (44:01**–**44:52)**—**Formulation phase**


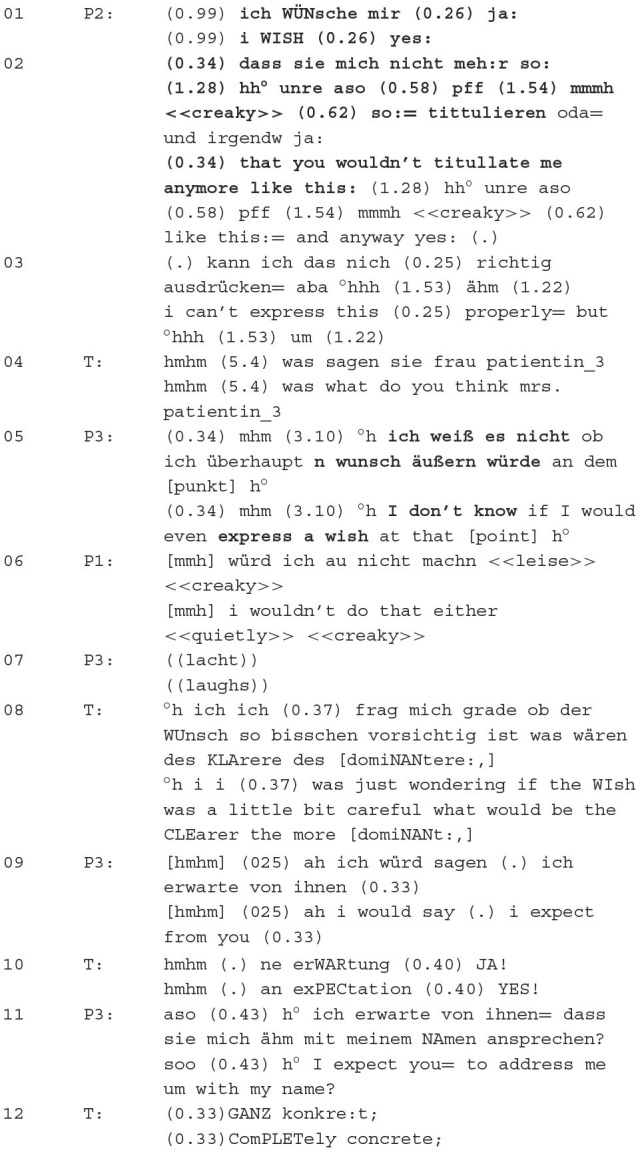


The context here is the writing of the message part, which is about the lack of verbalisation of P3's positioning to the salutation. P2 proposes the formulation *I WISH (0.26) yes: (0.34) that you wouldn't tittitulate me like that*. P3 then expresses her dispreference through an *I don't know* construction and positions herself, rejecting P2's suggestion (cf. Helmer et al., 2016; Helmer and Deppermann, 2017). P3 has established in the wish formulation phase that she wants to write a dominant and boundary-setting message. Thus, the wish does not fit her goals, which causes P3 to make a categorical evaluation of the positioning (categorisation practise; Torres Cajo, 2022, p. 70ff.). P1 follows this through *i wouldn't do that either*. The therapist responds to this categorisation and attributes the property carefully to the wish (attribution practise; Torres Cajo, 2022, p. 108ff.) and requests the category with more dominant properties. P3 then reformulates the wish into an expectation, which the therapist evaluates as an adequate formulation by saying *YES* (0.40) (evaluation practise; Torres Cajo, 2022, p. 142ff.). The patient then formulates a new sentence for the text message, which is evaluated as completely concrete by the therapist.

Here, the positionings serve the joint project, namely the writing of a message to the professor that is oriented towards the patient's wishes. In this way, proactive help is provided, the situation is worked through, and the patient is helped to increase her agency.

In a constant process of formulating text components as well as their acceptance, rejection, and reformulation, a draught message to the professor is created, which is oriented towards the wishes and goals established by Patient 3. The message is as follows:

“Dear Professor xy,

I consider the way of greeting inappropriate and disrespectful.

I expect you to address me by my name.

Hoping for a constructive Zoom meeting.

Until then,

best regards,

First name Last name Signature”

In the draught message, the patient positions herself in relation to the professor's inadequate form of address. She evaluates it as *inappropriate and disrespectful*, thus making up for her initial passivity and lack of reaction to the message. The evaluation with a corrective function is followed by a verbalised expectation with a limit-setting function. The professor is urged to address the patient only by her name in the future.

In response to the therapist's question about the evaluation of the message, Patient 3 answers: *(1.44) mmh (1.05) yes (1.46) good; (1.16) yes: in any case uh much better than uh (.) than being silent yes;*= *and I think that also sets another uh (1.61) uh hhh*° *ne another f form of uh (2.59) yes: of uh boundary and uh (1.22)* (52:24–52:48). The many hesitation signals, pauses and reformulations are striking in this utterance. These can be interpreted as reflection markers (cf. Gilquin, 2008, p. 120): P3 is in the process of feeling into herself and perceiving the transformation, which is supported by the various affirmations of the good feeling and the changed state (*yes, good, much better*). P3 is, therefore, reflecting and evaluating the current state in comparison to the initial state.

Patient 3 prefers the message to her silence and sees the response as setting a boundary, i.e., her goal of writing a dominant and boundary-setting message has been achieved. In the subsequent final phase of the conversation, the patients make it explicit that they have all learned something from this situation analysis.

Through the collaborative conception of this message draught, the group has co-constructed a counternarrative to Patient 3's actual response. In the protected setting of messenger-supported group psychotherapy, the situation can be re-enacted. Namely, the interactants pretend to write to the professor and rebuke him for his misbehaviour through the positioning. Through the support of the group and experimentation with different formulations, the stressful situation can be hypothetically worked through, co-constructing change in relationship and agency. This is because the patients not only have a changed possibility of a reaction, i.e., increased agency, but can also fall back on the solution path of this interaction situation and rely on the solidarity of the group. Therefore, transformation can be observed here on different levels: Transformation here concerns the response, the reaction, the ability to act, and the relationship to the group through the clear identification of a person in the outgroup, i.e., through the expression of positioning. The transformation also concerns the manageability of the conflict from “being alone with the problem” to “solving it together”. Through the exploration of the transformation and its authentic reporting, it can be concluded that a psychological change has taken place as a result of re-enacting the conflict.

## 4 Conclusion

The patients in eSA group psychotherapy use different practises for positioning. Categorisation, evaluation, attribution, narrative, and authentication practises could be identified. With regard to the different phases of the conversation, the positioning practises differ from each other: In the phase of describing the situation, categorisation, attribution, and narrative practises can be identified. This phase is characterised by the exchange of knowledge between the interactants. Here, the positionings primarily serve to classify the conflict and to describe the relationship to the other person. The phase of classifying one's own behaviour in the Kiesler circle is predominantly characterised by evaluation practises: The patient's behaviour is evaluated by means of given adjectives and verified by the group, which functions to raise awareness of one's own behaviour. Here, the patients are supposed to define an actual state before they formulate goals and wishes about their own behaviour, which is the basic component for the change of (maladaptive) behaviour. The formulation phase also mainly involves evaluation practises and refers to the formulation suggestions or the behaviour of the professor. The group successively formulates and reformulates text element by text element. In this process, the interactants are guided by the previously established goals and wishes of the patient concerned. The evaluative activities that refer to the professor's behaviour can be differentiated according to whether they remain internal to the group or are to be included in the message. This is because they also differ from each other functionally: Intra-group evaluations express solidarity with the patient and are thus group identity and relationship constituting. Evaluations in the message have the function of setting a limit to the professor and confronting his behaviour. In this way, these evaluations help patients to verbalise themselves and increase agency.

Relationship management in eSA group psychotherapy can, thus, be observed on the following different levels: (1) among the interactants in the room through the help provided in the form of solidarity and the formulation suggestions and (2) with the persons from the chat messages through the working on the common conflict. The patients can send text messages together with the positioning they contain and process the conflict in this way. The third level concerns the relationship between the patient(s) and the therapist. The therapist, by institutional format, takes a leading role in the interaction and provides the space for group formation and relationship building through the therapy concept and the specific successive steps. She moderates the group through all phases of conversation; she agrees, expresses compassion and intervenes. In addition, through her initial and tone-setting positioning towards the presented conflict, she enables the other patients to take further (sometimes extreme) positions, such as “because he was disGUsting”. The therapist supports P3 in classifying her behaviour in the Kiesler circle and thus sets a decisive milestone in the process of change: By becoming aware of an actual state and comparing it with a target state, change can be recorded in the first place. Through her final questioning, she supports the patient in noticing the transformation she has achieved. In addition, she intervenes for the purpose of formulating an adequate—in the sense of 'matching the patient's goals'—message, thereby providing proactive help. Through this work on common goals, she contributes significantly to the therapeutic alliance.

The interactants position themselves through evaluative adjectives, predication, laughter, or questions. In doing so, they refer to categorical characteristics of the conflict situation, evaluate their own behaviour or the behaviour of the professor, and formulate suggestions. Through positioning, implemented in narratives, interactants indicate how they relate to the persons in the messages and how they evaluate their behaviour. Such evaluations are perceived and understood by the interactants, so that in this way the possibility of expressing solidarity arises. By expressing solidarity in positionings, the group supports the patient, which contributes to the constitution of relationships and the formation of group identity. Furthermore, positionings are central in the collaborative formulation of a message. Because through them, formulation suggestions are accepted, rejected or reformulated. In this way, the interactants actively provide help.

With regard to transforming relations, the following can be concluded: A sense of unity develops between the group members, which is triggered by the distancing from the professor, i.e., “us against the professor”. The affected patient thus no longer feels alone with her problem. She is supported by the whole group. Furthermore, the whole group benefits from the exercise of solving a conflict in written form. This is because all those involved in the formulation can also refer back to the solution outside the session, so that their relationship to conflictual situations can change due to the increased ability to act. In addition, the conflict can be worked through in the various phases of group therapy, so that their attitude and feelings towards the conflict can also change as a result. Through the collaborative processing of the conflict, the solidarity of the group, and a concrete solution (including the way to it), the patient is supported in messenger-supported group psychotherapy to increase her ability to act.

In relation to the transference hypothesis of CBASP, it can also be stated that the patient's painful experience is consciously repeated in group psychotherapy, but with a different authority this time, namely the therapist. By re-enacting the painful experience in a new setting, the patient realises that different people can react differently in the same situation. The expectation of the transference hypothesis, namely that the difficult situation will also be repeated with other people or authorities, does not occur, so that the negative old experience can be overwritten by the positive new experience in therapy.

The eSA is thus an innovative psychotherapeutic format at the interface of therapy and writing counselling, through which knowledge is generated, relationships are shaped, and in this way, change is co-constructed. People with depression learn in eSA group psychotherapy to deal with other people in a more self-determined way and to experience new (positive) relationships.

## Data availability statement

The raw data supporting the conclusions of this article will be made available by the authors, without undue reservation.

## Ethics statement

The studies involving humans were approved by Ethics Committee of the LMU Munich. The studies were conducted in accordance with the local legislation and institutional requirements. Written informed consent for participation in this study was provided by the participants' legal guardians/next of kin. Written informed consent was obtained from the individual(s) for the publication of any potentially identifiable images or data included in this article.

## Author contributions

SK: Investigation, Writing—original draft.

## Footnotes

^1^I would like to express my sincere gratitude to Prof. Dr. Frank Padberg's team at LMU Munich and especially to Dr. rer. nat. Fabienne Große Wentrup for numerous inspiring discussions and the always excellent cooperation.
